# Gender difference in the incidence of malaria diagnosed at public health facilities in Uganda

**DOI:** 10.1186/s12936-022-04046-4

**Published:** 2022-01-21

**Authors:** Jaffer Okiring, Adrienne Epstein, Jane F. Namuganga, Emmanuel V. Kamya, Isaiah Nabende, Martha Nassali, Asadu Sserwanga, Samuel Gonahasa, Mercy Muwema, Steven M. Kiwuwa, Sarah G. Staedke, Moses R. Kamya, Joaniter I. Nankabirwa, Jessica Briggs, Prasanna Jagannathan, Grant Dorsey

**Affiliations:** 1 Clinical Epidemiology Unit, School of Medicine, Makerere University College of Health Sciences, Kampala, Uganda; 2 Infectious Diseases Research Collaboration, 2C Nakasero Hill Road, Kampala, Uganda; 3 Department of Epidemiology and Biostatistics, University of California San Francisco, San Francisco, USA; 4 Department of Child Health and Development Centre, School of Medicine, Makerere University College of Health Sciences, Kampala, Uganda; 5 London School of Hygiene and Tropical Medicine, Keppel Street, London, WC1E 7HT UK; 6 School of Medicine, Makerere University College of Health Sciences, Kampala, Uganda; 7 Department of Medicine, University of California San Francisco, San Francisco, USA; 8 Division of Infectious Diseases and Geographic Medicine, Stanford University, Stanford, USA

**Keywords:** Gender, Age, Malaria, Incidence, Differences, Routine, Surveillance

## Abstract

**Background:**

Routine malaria surveillance data in Africa primarily come from public health facilities reporting to national health management information systems. Although information on gender is routinely collected from patients presenting to these health facilities, stratification of malaria surveillance data by gender is rarely done. This study evaluated gender difference among patients diagnosed with parasitological confirmed malaria at public health facilities in Uganda.

**Methods:**

This study utilized individual level patient data collected from January 2020 through April 2021 at 12 public health facilities in Uganda and cross-sectional surveys conducted in target areas around these facilities in April 2021. Associations between gender and the incidence of malaria and non-malarial visits captured at the health facilities from patients residing within the target areas were estimated using poisson regression models controlling for seasonality. Associations between gender and data on health-seeking behaviour from the cross-sectional surveys were estimated using poisson regression models controlling for seasonality.

**Results:**

Overall, incidence of malaria diagnosed per 1000 person years was 735 among females and 449 among males (IRR = 1.72, 95% CI 1.68–1.77, p < 0.001), with larger differences among those 15–39 years (IRR = 2.46, 95% CI 2.34–2.58, p < 0.001) and over 39 years (IRR = 2.26, 95% CI 2.05–2.50, p < 0.001) compared to those under 15 years (IRR = 1.46, 95% CI 1.41–1.50, p < 0.001). Female gender was also associated with a higher incidence of visits where malaria was not suspected (IRR = 1.77, 95% CI 1.71–1.83, p < 0.001), with a similar pattern across age strata. These associations were consistent across the 12 individual health centres. From the cross-sectional surveys, females were more likely than males to report fever in the past 2 weeks and seek care at the local health centre (7.5% vs. 4.7%, p = 0.001) with these associations significant for those 15–39 years (RR = 2.49, 95% CI 1.17–5.31, p = 0.018) and over 39 years (RR = 2.56, 95% CI 1.00–6.54, p = 0.049).

**Conclusions:**

Females disproportionately contribute to the burden of malaria diagnosed at public health facilities in Uganda, especially once they reach childbearing age. Contributing factors included more frequent visits to these facilities independent of malaria and a higher reported risk of seeking care at these facilities for febrile illnesses.

## Background

Over the past twenty years the scale-up of malaria control efforts has led to marked reductions in morbidity and mortality. However, global progress has slowed in recent years, particularly in the WHO African Region, which accounted for 94% of the world’s 229 million cases in 2019 [1]. Malaria surveillance is considered a core intervention and critical for the purposes of monitoring and evaluation, especially in African countries where the burden of malaria remains high [2]. The most widely available source of routine malaria surveillance data in Africa come from public health facilities reporting to national health management information systems (HMIS). Although information on gender is routinely collected from patients presenting to public health facilities, stratification of malaria surveillance data by gender is rarely done. Studies have suggested differences may exist between females and males in the risk of infection and disease, but few studies have evaluated gender differences in the context of routine malaria surveillance data from public health facilities [3, 4]. An appreciation of gender difference in malaria burden would be important for improving the understanding of factors that may influence susceptibility to malaria, case management practices, and targeting control interventions.

There are many potential factors that could contribute to differences in measures of malaria between females and males. Social, cultural, and behavioural differences may influence one’s risk of exposure to mosquito vectors, perception of illness, health-seeking behaviour, and case management practices [5–7]. Sex-specific factors, such as the relationship between malaria and pregnancy, have also been well described [8–10]. In addition, sex-related biological differences may influence the risk of becoming infected with malaria parasites, whether infection leads to clinical disease, and the ability to clear infections [11, 12]. These complex, multifactorial relationships suggest that associations between gender and malaria are likely to be modified by local epidemiological factors, demographics (i.e., age), and malaria outcomes being assessed (i.e., infection vs. disease).

Most data available on associations between gender and malaria come from cohort and cross- sectional studies[3, 4, 13, 14], which may not be representative of patients who seek care at public health facilities and contribute to routine malaria surveillance data through HMIS. In Uganda, an enhanced health facility-based malaria surveillance system has been established at sentinel public health centres to provide high quality data around the country. At these public health centres, individual patient level data are collected and resources provided to maximize laboratory testing for malaria and improve data quality. More recently, data on village of residence have been captured and targeted areas around a subset of public health centres identified, enumerated and surveyed, allowing for estimation of malaria incidence from within these target areas. This study evaluated associations between gender and the incidence of malaria diagnosed at 12 public health centres over a 16-month period.

## Methods

### Health facility-based malaria surveillance and study setting

This study leveraged data from the Uganda Malaria Surveillance Project (UMSP), which established a public health facility-based malaria surveillance system in collaboration with the Uganda National Malaria Control Division (NMCD) beginning in 2006. These public health facilities, referred to as Malaria Reference Centres (MRCs) are level III/IV health facilities that generally see between 1000 and 3000 outpatients per month and have functioning laboratories. Of note, the UMSP malaria surveillance system does not include level II health facilities due to a lack of laboratory facilities or private health facilities, which could have affected the generalizability of the study findings. At each MRC, individual-level data from standardized HMIS registers for all patients presenting to the outpatient departments are entered into an Access database by on-site data officers. These data have been described elsewhere [15] and briefly includes village of residence, age, gender, whether malaria was suspected, whether a malaria diagnostic test was done, the type of diagnostic test done (rapid diagnostic test (RDT) or microscopy), and the result of the diagnostic test (positive or negative). UMSP supports the sites with training, supervision, and buffer stock of laboratory supplies/consumables. Full-time regional surveillance assistants are based around the country; each supervising 8–10 MRCS. Site support supervision is conducted on a regular basis to provide refresher training on malaria case management, review data quality, and perform laboratory external quality control for malaria microscopy. Core UMSP team members are also responsible for generating periodic reports, communicating with Ministry of Health officials and other key stakeholders, and conducting data analyses.

This study included data collected from January 2020 through April 2021 from 12 MRCs which met the following criteria: (1) location in areas where indoor residual spraying of insecticide is not being implemented; (2) less than 5% missing data for each of the following variables; age (all patients), village of residence (all patients), and results for malaria diagnostic testing (among patients with suspected malaria); and (3) household enumeration and cross-sectional surveys conducted in April 2021 within target areas identified around each MRC [16]*.*

### Identification of MRC target areas

**Fig. 1:**
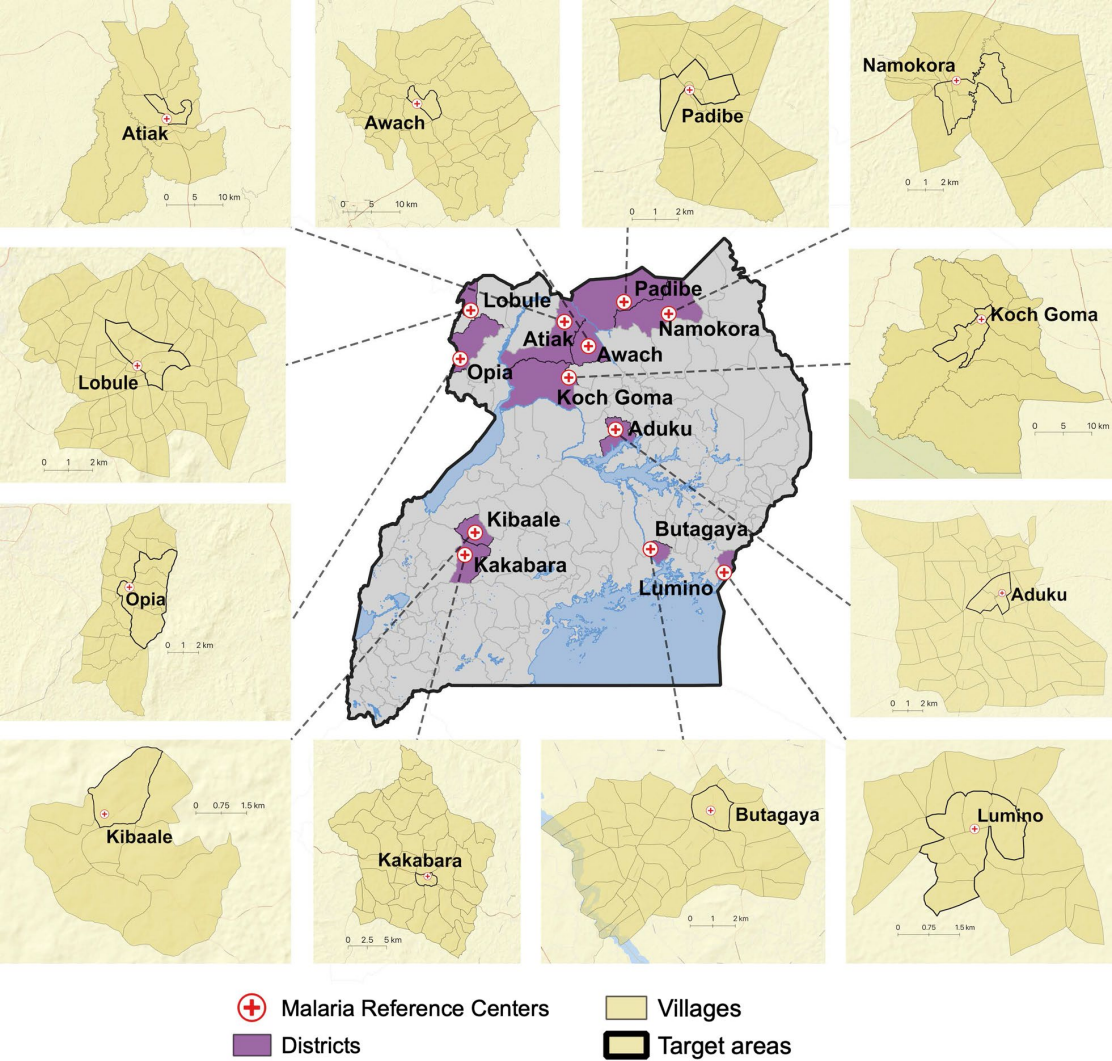
Map of Uganda showing the study districts, health facility target areas, and local public health facilities: Districts (purple shaded), health facility target areas (bold black with yellow filled color), and local public health facilities (bold red cross)

Target areas were identified around each MRC based on the assumption that the majority of patients within the target area who developed malaria would be captured by the health facility-based surveillance system. Target areas included the village where the MRC is located and adjacent villages that met all of the following criteria: (1) did not contain another public health facility, (2) were in the same sub-county where the MRC is located, (3) had a similar incidence of malaria as the village where the MRC is located. Target areas around each MRC included between 1 and 7 villages (Fig. 1).

### Target area household enumeration and cross-sectional surveys

All houses within the target area of each MRC were enumerated to facilitate population estimates and generate a sampling frame for cross-sectional surveys. Houses were approached in random order from the enumeration list and enrolled in the cross-sectional survey if all of the following criteria were met: (1) at least one adult aged 18 years or older present, (2) adult is a usual resident who slept in the sampled household on the night before the survey, and (3) agreement of the adult resident to provide informed consent. Prior to conducting the surveys, a Research Assistant briefed the household head or designate about the study, making it clear that participation was completely voluntary. For each MRC target area, consecutive eligible houses were surveyed until a total of 50 houses with at least 1 child aged 2–10 years were enrolled. Household resident level data from the cross-sectional surveys used for this study included age, gender, whether the resident reported fever in the past 2 weeks, and if fever was reported, whether care was sought at the local MRC.

### Population estimates of the MRC target areas

Estimates of the total population of the target area for each MRC for the month the surveys were conducted (April 2021) were generated using the following formula: (total number of residents from the houses surveyed/number of houses surveyed) × the total number of houses enumerated within the target area. Population in the preceding months were estimated using a fixed population growth function of 0.0029 per unit time [17]. MRC target area specific gender and age stratified population estimates were generated by multiplying the proportion of each gender and age strata from the population of houses surveyed by the total population (Table 1). Three age strata were selected a priori to reflect the periods before, during, and after the age range when fertility rates are > 100/1000 women according to the 2016 Uganda Demographic and Health Survey [18].

**Table 1:** Descriptive data from household enumeration and census survey from each MRC

MRC	Total number of houses enumerated	Number of houses surveyed	Population of houses surveyed	Gender and age strata from population of houses surveyed, n (% total)					Total population of the target area*
				Gender	All ages	Age strata			
						< 15 years	15–39 years	> 39 years	
Kakabara	581	57	271	Male	127 (46.9)	73 (26.9)	41 (15.1)	13 (4.8)	2762
				Female	144 (53.1)	72 (26.6)	58 (21.4)	14 (5.2)	
Kibaale	361	53	246	Male	109 (44.3)	63 (25.6)	36 (14.6)	10 (4.1)	1676
				Female	137 (55.7)	62 (25.2)	58 (23.6)	17 (6.9)	
Opia	667	54	299	Male	151 (50.5)	73 (24.4)	58 (19.4)	20 (6.7)	3693
				Female	148 (49.5)	73 (24.4)	58 (19.4)	17 (5.7)	
Lobule	260	51	299	Male	175 (58.5)	99 (33.1)	44 (14.7)	32 (10.7)	1524
				Female	124 (41.5)	62 (20.7)	48 (16.1)	14 (4.7)	
Koch Goma	1049	50	238	Male	113 (47.5)	70 (29.4)	33 (13.9)	10 (4.2)	4993
				Female	125 (52.5)	58 (24.4)	52 (21.9)	15 (6.3)	
Atiak	726	62	312	Male	153 (49.0)	85 (27.2)	49 (15.7)	19 (6.1)	3653
				Female	159 (51.0)	73 (23.4)	53 (17.0)	33 (10.6)	
Awach	620	52	232	Male	103 (44.4)	48 (20.7)	33 (14.2)	22 (9.5)	2766
				Female	129 (55.6)	64 (27.6)	39 (16.8)	26 (11.2)	
Padibe	773	59	296	Male	133(44.9)	61 (20.6)	53 (17.9)	19 (6.4)	3878
				Female	163 (55.1)	76 (25.7)	67 (22.6)	20 (6.8)	
Namokora	373	53	275	Male	126 (45.8)	66 (24.0)	43 (15.6)	17 (6.2)	1935
				Female	149 (54.2)	76 (27.6)	52 (18.9)	21 (7.6)	
Aduku	303	63	241	Male	103 (42.7)	52 (21.6)	29 (12.0)	22 (9.1)	1159
				Female	138 (57.3)	64 (26.6)	45 (18.7)	29 (12.0)	
Butagaya	251	50	339	Male	165 (48.7)	91 (26.8)	50 (14.8)	24 (7.1)	1702
				Female	174 (51.3)	87 (25.7)	51 (15.0)	36 (10.6)	
Lumino	1070	55	294	Male	126 (42.9)	61 (20.8)	42 (14.3)	23 (7.8)	5720
				Female	168 (57.1)	88 (29.9)	59 (20.1)	21 (7.1)	

*Total population of the target area = (population of houses surveyed/number of houses surveyed) × total number of houses enumerated

### Outcome measures derived from surveillance data collected at the MRCs

Suspected malaria was defined as all patients referred for malaria diagnostic testing plus all patients not referred for diagnostic testing but were given a clinical diagnosis of malaria. Test positivity rate was defined as the proportion of all patients tested for malaria who tested positive. Parasitological confirmed malaria was defined as any patient with a positive diagnostic test (RDT or microscopy) for malaria. Incidence of malaria diagnosed at the MRCs was defined as the total number of parasitological confirmed cases of malaria diagnosed over the 16 month study period at the MRCs from patients residing in villages within the target areas divided by the total person time observed from the total population of the target areas. Incidence of visits with malaria not suspected was defined as the total number of visits among patients not referred for diagnostic testing or given a clinical diagnosis of malaria over the 16 month study period at the MRCs from patients residing in villages within the target areas divided by the total person time observed from the total population of the target areas.

### Statistical analysis

Data were analysed using Stata version 14.1 (College Station, TX) and R software version 3.6.0. For analyses of measures of malaria case management and incidence, data were collapsed by month of observation for each individual MRC. Measures of malaria case management included the (1) proportion of total visits with malaria suspected, (2) proportion of visits with suspected malaria where a diagnostic test was done, (3) proportion of diagnostic tests where an RDT was performed, and (4) proportion of diagnostic tests that were positive for malaria (commonly referred to as the test positivity rate). Associations between gender and measures of malaria case management were estimated using generalized linear models controlling for calendar month with robust standard errors and random effects for study site. Site specific associations between gender and incidence measures (incidence of malaria diagnosed at the MRCs and incidence of visits to the MRCs with malaria not suspected) were expressed as incidence rate ratios (IRRs) and estimated using poisson regression models controlling for calendar month with population estimates as an offset. Random effects for study site was also included when combining data across all 12 MRCs. Analyses of binary outcomes of health-seeking behaviour from the cross-sectional surveys (reported fever in the past 2 weeks and reported fever in the past 2 weeks with care sought at the local MRC) were only conducted when combining data across all 12 MRCs as there were insufficient data to conduct analyses stratified by study site. Associations between gender and binary outcomes measures were expressed as risk ratios (RRs) and estimated using poisson regression models controlling for calendar month with robust standard errors and random effects for study site. All analyses were also stratified by three age categories determined a priori as described above. A two-side p-value of < 0.05 was considered statistically significant. The number of sites and houses included in the cross-sectional surveys were not based on any sample size calculations but rather by convenience and resources available.

## Results

### Summary description of target area population estimates

A total of 7034 houses were enumerated within the target areas of all 12 MRCs (range 251–1070 per MRC). A total of 659 houses were surveyed (range 50–63 per MRC) and 3342 household members identified (range 232–339 per MRC). The total population within the target areas of all 12 MRCs was estimated to be 35,461 (range 1159–4933 per MRC). Among the study population of all houses surveyed, 52.6% were female (range 41.5–57.3% across the MRCs) and 50.8%, 34.4% and 14.8% were under 15 years of age, 15–39 years of age, and over 39 years of age, respectively. The proportion of females was similar across the 3 age strata (Table 1).

### Associations between gender and measures of malaria case management at the MRCs

There were a total of 60,461 outpatient visits among patients residing in the target areas of the 12 MRCs over the 16 month observation period (Table 2). Over twice as many visits were among females compared to males (40,847 vs. 19,614). Clinic visits among females were greater than males across all age strata, with the greatest difference among patients 15–39 years of age (18,652 vs. 5430 visits). Overall, a similarly high proportion of females and males were suspected of having malaria (71.9% vs. 71.0%, p = 0.83). However, when stratified by age, females were more likely to have suspected malaria than males among patients 15–39 years of age (66.6% vs. 60.2%, p < 0.001) and > 39 years of age (57.2% vs. 46.3%, p < 0.001). Among patients with suspected malaria, over 99% had a diagnostic test performed with no significant differences between females and males. Among those tested for malaria, over 92% had an RDT done (as opposed to microscopy) with no significant differences between females and males. Among those tested for malaria, the overall test positivity was higher in males compared to females (69.9% vs. 61.8%, p < 0.001). However, when stratified by age, these differences were only significant among patients 15–39 year of age (67.2% vs. 55.2%, p < 0.001) and > 39 years of age (52.5% vs. 41.8%, p < 0.001).

**Table 2:** Health facility-based data stratified by age and gender from patients residing in target areas of all MRCs combined

Age strata	Gender	Total outpatient visits	Visits with suspected malaria (% total visits)	Diagnostic test performed* (% with suspected malaria)	RDT performed (% tested)	Parasitological confirmed malaria (% tested)
All ages	Male	19,614	13,934 (71.0)	13,907 (99.8)	12,760 (91.8)	9,726 (69.9)
	Female	40,847	29,381 (71.9)	29,321 (99.8)	27,258 (93.0)	18,107 (61.8)
< 15 years	Male	11,787	9,552 (80.7)	9,535 (99.8)	8785 (92.1)	6,949 (73.9)
	Female	16,500	13,709 (83.1)	13,681 (99.8)	12,671 (92.6)	9906 (72.4)
15–39 years	Male	5430	3271 (60.2)	3265 (99.8)	2959 (90.6)	2195 (67.2)
	Female	18,652	12,415 (66.6)	12,395 (99.8)	11,579 (93.4)	6845 (55.2)
> 39 years	Male	2397	1111 (46.3)	1107 (99.6)	1016 (91.8)	582 (52.5)
	Female	5695	3257 (57.2)	3245 (99.6)	3008 (92.7)	1356 (41.8)

*Either RDT or light microscopy

### Associations between gender and incidence measures diagnosed at the MRCs

**Fig. 2:**
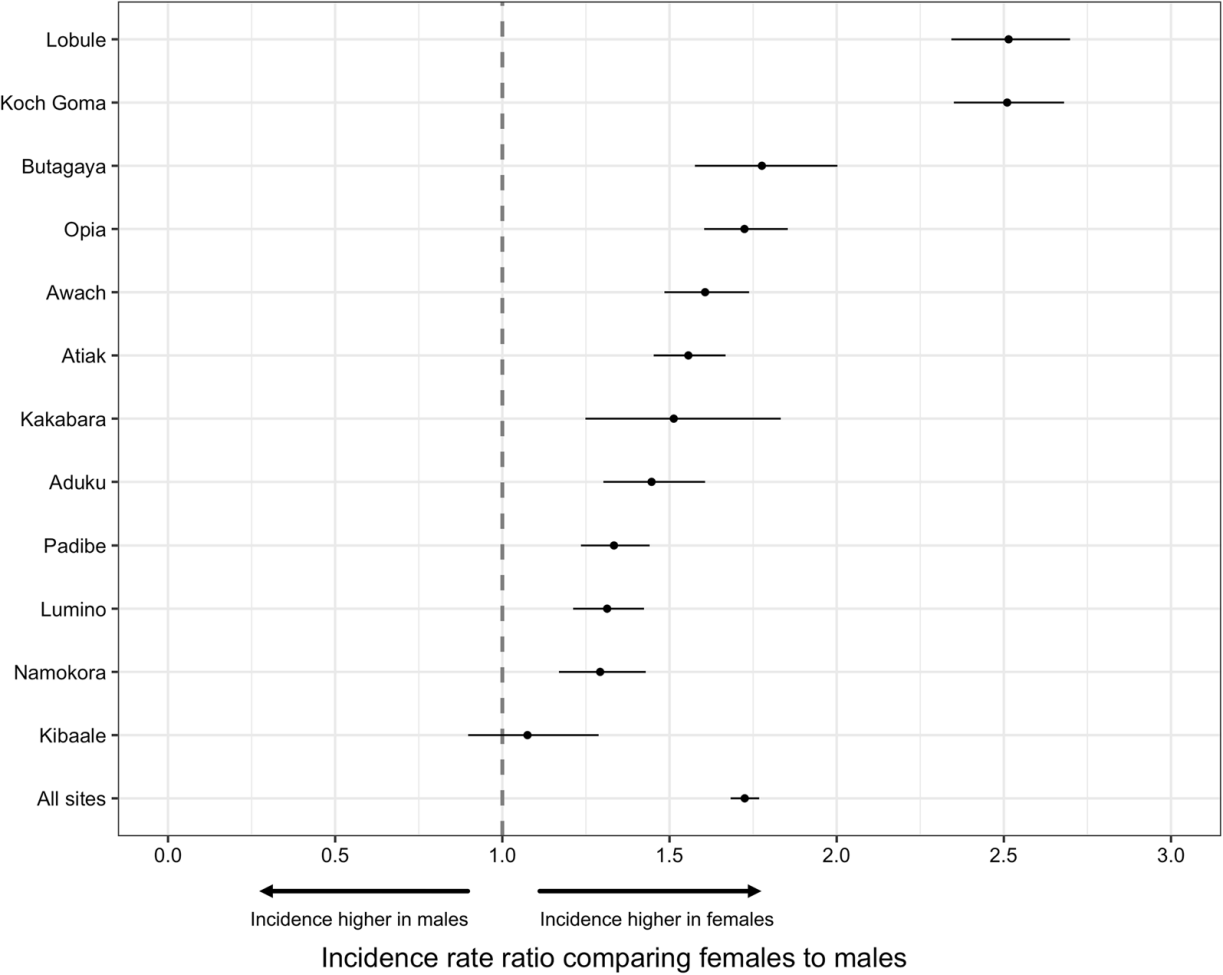
A forest plot showing incidence rate ratio of malaria diagnosed among female gender relative to males at each local public health facility and all sites combined (block vertical dotted line indicates the null hypothesis; incidence of malaria diagnosed is not different across gender)

There were a total of 27,833 visits with parasitological confirmed malaria among patients residing in the target areas of the 12 MRCs over the 16 month observation period (Table 3). Almost twice as many visits with malaria diagnosed were among females compared to males (18,107 vs. 9726). When accounting for the estimated gender stratified populations of the target areas, the incidence of malaria diagnosed per 1000 person years for all 12 MRCs combined was 735 among females and 449 among males (IRR = 1.72, 95% CI 1.68–1.77, p < 0.001). Female gender was associated with a higher incidence of malaria across all individual MRCs, although the magnitude of these associations varied with IRRs ranging from 1.08 to 2.51 (Fig. 2). As expected, the incidence of malaria diagnosed at the MRCs decreased with increasing age. The magnitude of the association between female gender and malaria incidence was higher among those 15–39 years of age (IRR = 2.46, 95% CI 2.34–2.58, p < 0.001) and over 39 years of age (IRR = 2.26, 95% CI 2.05–2.50, p < 0.001) compared to those under 15 years of age (IRR = 1.46, 95% CI 1.41–1.50, p < 0.001) (Table 3).

**Table 3:** Associations between gender and incidence of malaria diagnosed at the MRCs from the target areas of all MRCs combined

Age strata	Gender	Visits with malaria diagnosed	Person years of observation	Incidence per 1000 PY	IRR (95% CI)*	p-value
All ages	Male	9726	21,654	449	Reference group	
	Female	18,107	24,638	735	1.72 (1.68–1.77)	< 0.001
< 15 years	Male	6949	11,452	607	Reference group	
	Female	9906	12,005	825	1.46 (1.41–1.50)	< 0.001
15–39 years	Male	2195	7137	308	Reference group	
	Female	6845	9122	750	2.46 (2.34–2.58)	< 0.001
> 39 years	Male	582	3065	190	Reference group	
	Female	1356	3511	386	2.26 (2.05–2.50)	< 0.001

^*^ adjusted for calendar month and clustering at the level of the MRC

**Fig. 3:**
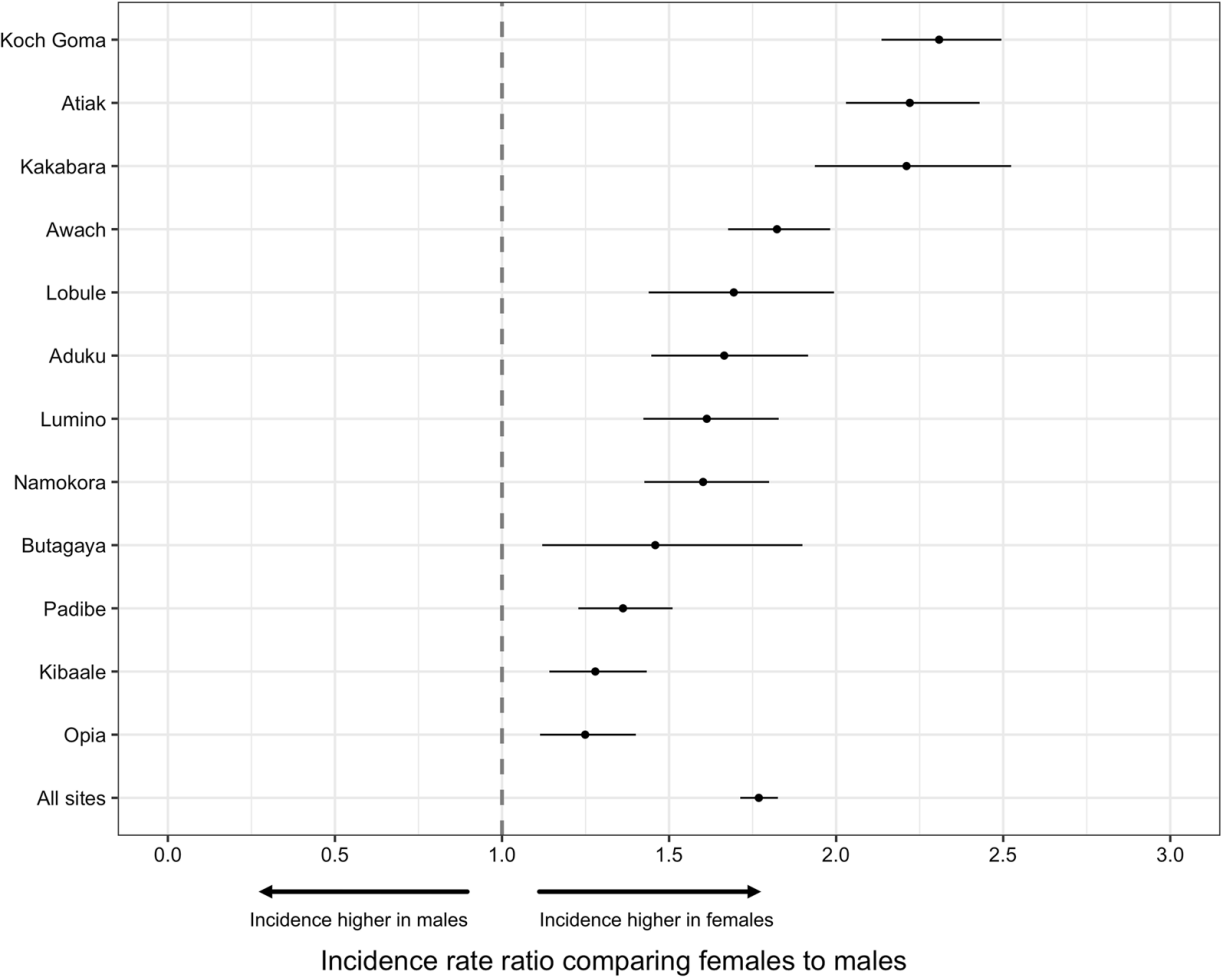
A forest plot showing incidence rate ratio of visits with malaria not suspected among female gender relative to males at each local public health facility and all sites combined (block vertical dotted line indicates the null hypothesis; incidence of visits with malaria not suspected is not different across gender)

To evaluate utilization of the MRCs independent of malaria, associations between gender and the incidence of visits with malaria not suspected were assessed (Table 4). Considering all ages, female gender was associated with a higher incidence of visits with malaria not suspected (IRR = 1.77, 95% CI 1.71–1.83, p < 0.001) for all 12 MRCs combined as well as for each individual MRC (Fig. 3). When stratified by age, associations between female gender and the incidence of visits with malaria not suspected followed a similar pattern as associations between female gender and the incidence of malaria, although the magnitude of these associations were not as great (Table 4).

**Table 4:** Associations between gender and incidence of visits with malaria not suspected from the target areas of all MRCs combined

Age strata	Gender	Visits with malaria not suspected	Person years of observation	Incidence per 1000 PY	IRR (95% CI)*	p-value
All ages	Male	5680	21,654	262	Reference group	
	Female	11,466	24,638	465	1.77 (1.71–1.83)	< 0.001
< 15 years	Male	2235	11,452	195	Reference group	
	Female	2791	12,005	232	1.22 (1.16–1.29)	< 0.001
15–39 years	Male	2159	7137	303	Reference group	
	Female	6237	9122	684	2.23 (2.12–2.34)	< 0.001
> 39 years	Male	1286	3065	420	Reference group	
	Female	2438	3511	694	1.57 (1.47–1.68)	< 0.001

*Adjusted for calendar month and clustering at the level of the MRC

### Associations between gender and health-seeking behaviour from cross-sectional surveys for all MRCs combined

To further explore differences in health-seeking behaviour between females and males, associations between gender and questions about recent fever were assessed using data collected from cross-sectional surveys conducted among randomly selected houses within the target areas of all 12 MRCs. At total of 3342 household members were included in the surveys including 1758 females and 1584 males (Table 5). Considering all ages, a higher proportion of females compared to males (10.8% vs. 7.5%) reported fever in the past 2 weeks (RR = 1.40, 95% CI 1.13–1.74, p = 0.002). When stratified by age, associations between female gender and a higher risk of reported fever the past 2 weeks (Table 6) were only significant for those over 39 years of age (RR = 2.56, 95% CI 1.10–5.95, p = 0.029). Among those who reported fever in the past 2 weeks, the proportion who sought care at the local MRC was similar between females and males with the exception of those 15–39 years of age, where it was higher among females (76.9% vs. 52.0%). The risk of reporting a fever in the past 2 weeks and seeking care at the local MRC was higher in females compared to males (Table 6), but these associations were only significant for those 15–39 years of age (RR = 2.49, 95% CI 1.17–5.31, p = 0.018) and over 39 years of age (RR = 2.56, 95% CI 1.00–6.54, p = 0.049).

**Table 5:** Characteristics of residents included in the cross-sectional surveys stratified by gender

Characteristic	Category	Findings, n (%)	
		Male (n = 1584)	Female (n = 1758)
Age	Under 15 years	842 (53.2)	855 (48.6)
	15–39 years	511 (32.3)	640 (36.4)
	Over 39 years	231 (14.5)	263 (15.0)
Relationship to head of household	Head of household	433 (27.3)	201 (11.4)
	1st degree relative	894 (56.5)	1326 (75.4)
	2nd degree/unrelated	257 (16.2	231 (13.2)
Reported sleeping under a bed net the prior evening		1226 (77.4)	1390 (79.1)
Reported being treated for malaria in the prior 1 month		305 (19.3)	360 (20.5)
Reported fever in the prior 2 weeks		119 (7.5)	189 (10.8)
Where care sought if reported fever in the prior 2 weeks	Local MRC	75 (63.0)	131 (69.3)
	Other public health facility	1 (0.8)	4 (2.1)
	Private sector	31 (26.1)	37 (19.6)
	Other/care not sought	12 (10.1)	17 (9.0)

**Table 6:** Associations between gender and health-seeking behaviour from cross-sectional surveys for all MRCs combined

Age strata	Gender	Number surveyed	Reported fever in the past 2 weeks	RR (95% CI)	p-value	Reported fever in the past 2 weeks and sought care at local MRC	RR (95% CI)	p-value
All ages	Male	1584	119 (7.5)	Reference group		75 (4.7)	Reference group	
	Female	1758	189 (10.8)	1.40 (1.13–1.74)	0.002	131 (7.5)	1.56 (1.21–2.02)	0.001
< 15 years	Male	842	83 (9.9)	Reference group		55 (6.5)	Reference group	
	Female	855	104 (12.2)	1.19 (0.96–1.48)	0.108	70 (8.2)	1.22 (0.93–1.60)	0.147
15–39 years	Male	511	25 (4.9)	Reference group		13 (2.5)	Reference group	
	Female	640	52 (8.1)	1.66 (0.85–3.23)	0.136	40 (6.3)	2.49 (1.17–5.31)	0.018
> 39 years	Male	231	11 (4.8)	Reference group		7 (3.0)	Reference group	
	Female	263	33 (12.5)	2.56 (1.10–5.95)	0.029	21 (8.0)	2.56 (1.00–6.54)	0.049

## Discussion

This study utilized data collected from 12 public health facilities in Uganda over a 16 month period to evaluate whether the burden of parasitological confirmed malaria differed between females and males. Females accounted for almost twice as many cases of malaria diagnosed at these facilities compared to males. When adjusting for gender stratified population estimates in target areas around the health facilities, the incidence of malaria diagnosed at these facilities was over 70% higher in females. Furthermore, this association was modified by age. Considering those 15 years and older, females had over twice the incidence of malaria diagnosed at these facilities compared to males. In contrast, incidence was less than 50% higher in females when considering children under 15 years of age. Additional data were utilized to explore possible explanations for the excess burden of malaria diagnosed at these facilities among females, particularly among adults. Based on community surveys, females 15 years and older were over twice as likely as males to report visiting their local health facility for recent fever. At the health facilities, females 15 years and older were slightly more likely than males to have malaria suspected, but there was no difference in diagnostic testing practices between females and males. Interestingly, among those tested for malaria, males 15 years and older had a modestly higher risk of testing positive compared to females. Finally, the incidence of visits to the health facilities when malaria was not suspected was higher in females compared to males, particularly among those over 15 years of age.

The primary objective of the study in this report was to evaluate gender differences among patients diagnosed with parasitological confirmed malaria at public health facilities in Uganda. Although data on gender is often collected from patients presenting to public health facilities, routine malaria surveillance data is rarely disaggregated by gender. Given prior studies suggesting that *Plasmodium* parasite prevalence may be higher in post-adolescent males vs. females [19–22]; it was somewhat surprising that the burden of malaria at these health facilities was so much higher in adult females compared to males, especially after typical childbearing age (i.e., above 39 years of age). Additional data provided in this study helped shed light on factors that may explain this difference, although many questions could not be addressed. From cross-sectional surveys in communities around the health facilities, adult females were much more likely than males to report recent fever and seek care at the local health facility, although there was insufficient data to disentangle the relative contribution of recent fever vs. where care was sought. Similarly, at the health facilities a markedly higher number of patients with suspected malaria were females compared to males, especially among adults. At the same time, females were also more likely than males to visit the health facilities when malaria was not suspected. Thus, it appears likely that females utilize public health facilities more often than males regardless of whether or not they have symptoms of malaria. This could be due to the fact that in most parts of the world females have the primary responsibility of caring for others in the household, in addition to potentially being pregnant, and may end up being “coincidentally” tested and diagnosed for malaria when their primary reason for visiting the health facility was either to seek care for a child who was ill or attend antenatal care. However, this is only speculative as in this study we did not have access to data on why patients chose to attend the health facilities. Once patients presented to the health facilities with suspected malaria, there were no gender differences in the probability of getting tested for malaria or the type of diagnostic test done.

This study also found that, among those tested, post-adolescent males had a higher probability than females of testing positive. This is consistent with other studies conducted in both hyperendemic and hypoendemic areas outside of Africa [19–25] which have also observed a male bias in parasite prevalence among adolescents and adults. This male bias in parasite prevalence has been attributed to a number of factors, including females of reproductive age being more likely to sleep under a bed net, behavioural differences in alcohol and tobacco consumption leading to increased male attractiveness to mosquitoes, and sex-specific biologic differences, including post-pubertal hormonal changes [26–28]. Importantly, differences in parasite prevalence between the sexes may be either due to differences in infection incidence or in the duration of individual infections. In a recent study from an area of Uganda where transmission intensity had been markedly reduced following highly effective vector control intervention, a cohort of all members of 80 households were followed for 2 years with passive surveillance for symptomatic malaria and active surveillance every 28 days for parasitaemia using an ultrasensitve quantitative PCR assay and amplicon deep sequencing to distinguish persistent from new infections [29].

In this study, there was no sex-specific difference in the molecular force of infection (number of new infections per time), but parasite prevalence was higher in males compared to females (2.9 vs. 1.4%) and males had a longer duration of infection [4]. Interestingly, the incidence of symptomatic malaria for participants over 10 years of age was over twice as high in females compared to males, although malaria was uncommon in this cohort and the difference between females and males was not statistically significant (37 vs. 18 episodes per 1000 person years, p = 0.22). This suggests that although males have longer duration infections, females might have a higher probability of symptoms given an infection. Supporting this hypothesis, in another recent cohort study from Western Kenya, individuals with asymptomatic parasitaemia had an increased 1-month likelihood of symptomatic malaria compared to those who were uninfected; importantly, these associations were over twice as strong in females compared to males with adjusted Hazard Ratios of 3.71 vs. 1.76 [30]. Collectively, these studies suggest that sex-specific differences may exist in naturally acquired immunity to malaria, with males being less able to control parasite densities (anti-parasite immunity), leading to higher parasite prevalence among males, and females being less able to tolerate higher parasite densities without fever (anti-disease immunity) [31], leading to a higher probability of symptoms once infected among females.

This study had several limitations. First, parasitaemia was not assessed in the communities around the health facilities and therefore it was not possible to evaluate for associations between gender and the risk of asymptomatic parasitaemia or the risk of symptomatic malaria when parasitaemic. Second, data on whether woman presenting to the health facilities were pregnant was not available, although associations between female gender and an increased burden of malaria were generally strongest for the age strata when fertility rates are highest. Numerous studies have demonstrated that pregnant women are at increased risk of *P. falciparum* infection and experience higher parasite densities and rates of clinical malaria than non-pregnant women [32–35]. Third, estimates of malaria incidence were based on cases diagnosed at the health facilities and therefore did not include episodes of malaria that were not captured by the health facility-based surveillance system. Forth, the diagnosis of malaria was primarily based on the results of RDTs, which may have relatively low specificity in high transmission settings [36]. Fifth, data were limited to 12 level III/IV public health facilities in predominantly rural areas of Uganda where the burden of malaria is high and therefore caution should be taken when generalizing findings to other epidemiological settings. Sixth, this study was largely conducted following the onset of the COVID-19 pandemic, which could have affected health-seeking behaviour. However, a recent study utilizing data from the same health-facility based malaria surveillance system reported no changes in the total outpatient visits, malaria cases, non-malarial visits, or proportion of visits with suspected malaria when comparing data from three years prior to the onset of the COVID-19 pandemic in Uganda to one year after the onset of the COVID-19 pandemic [37].

Finally, the study did not assess biological factors that may have provided additional information on mechanisms to explain differences in malaria burden between females and males [38–40]. Despite these limitations, the large samples size, magnitude of the differences seen, and the consistency of findings across many different study sites supports the robustness of the main study findings.

## Conclusion

In this study, a relatively novel approach was used to estimate malaria incidence in target communities around public health centres using routinely collected data. The incidence of malaria diagnosed at these pubic health facilities was higher among females compared to males, with more than a twofold increase among persons 15 years or older. Females were also more likely to visit these facilities independent of malaria and were more likely to report seeking care at these facilities for febrile illnesses. These finding have practical implications, including a better understanding of the role of gender in health care utilization and supports the targeting of women. For example, women attending public health facilities (including antenatal clinics) could be provided LLINs and educated on other ways to prevent malaria. Malaria surveillance activities should routinely disaggregate data on gender and future studies are needed to better understand biological and socio-behavioural factors that may explain gender-specific differences in the complex interplay between malaria transmission, infection, and disease.

## Data Availability

The datasets used for this study are available from the corresponding author on reasonable request.

## Abbreviations

ACT: Artemisinin-based combination therapy
HC: Health centre
HMIS: Health Management Information Systems
IPT: Intermittent preventive therapy
IRR: Incidence rate ratio
IRS: Indoor residual spraying
MRC: Malaria reference centre
NMCD: National Malaria Control Division
PM: Placenta malaria
TPR: Test positivity rate
RDT: Rapid diagnostic test
RR: Risk ratio
UMSP: Uganda Malaria Surveillance Project
WHO: World Health Organization
